# Remote work and long-term sickness absence due to mental disorder trends among Japanese workers pre/post COVID-19

**DOI:** 10.1371/journal.pone.0319825

**Published:** 2025-03-12

**Authors:** Yasuhiko Deguchi, Shinichi Iwasaki, Yuki Uesaka, Yutaro Okawa, Shohei Okura, Kunio Maekubo, Ayaka Matsunaga, Yuki Kageyama, Koki Inoue

**Affiliations:** 1 Department of Neuropsychiatry, Osaka Metropolitan University Graduate School of Medicine, Osaka, Japan; 2 Osaka Occupational Health Support Center, Osaka, Japan; University of Jyvaskyla, FINLAND

## Abstract

**Aims:**

The aim of this study was to ascertain whether there has been an increase in the number of workers with long-term sickness absence due to mental disorders (LTSA-MD) and determine the impact of remote work on new LTSA-MD cases.

**Methods:**

A web-based questionnaire was sent to 2,552 company offices with 150 or more workers in Osaka Prefecture. Data were obtained on the number of workers with LTSA-MD between April 1, 2019, and March 31, 2020 (fiscal year 2019) and between April 1, 2020, and March 31, 2021 (fiscal year 2020), along with their MD diagnoses (adjustment disorder [AD], depressive disorder [DEP], etc.). The difference in the number of new LTSA-MD, LTSA-AD, and LTSA-DEP cases between the fiscal years was evaluated, as well as the number of LTSA-MD cases per 100 employees. An independent t-test was used to compare the groups.

**Results:**

DEP was the most prevalent condition, followed by AD. The number of workers with LTSA-MD nominally decreased from fiscal 2019 to fiscal year 2020, with no significant difference. There were no significant differences between fiscal year 2020 and fiscal year 2019 regarding LTSA-MD, LTSA-AD, and LTSA-DEP in offices with and without a remote work model.

**Conclusions:**

The number of non-public workers with LTSA-MD did not increase during the COVID-19 pandemic, with no significant difference observed between offices with and without a remote work model. This provides preliminary evidence of a potential protective effect of remote work against LTSA-DEP.

## Introduction

An estimated one in three individuals will experience a mental disorder (MD) during their lifetime [1–3]. Mental health conditions ranked among the top 10 major global burdens in 2019 [4] and were revealed to be costly for societies worldwide [5]. MDs profoundly impact affected individuals, their coworkers, families, and society at large through increased healthcare costs [6], shortened healthy life expectancy [7], loss of human resources [6]. Additionally, MDs often lead to long-term sickness absence (LTSA) from work [8–10], which is defined in this study as a period of sickness absence lasting 30 days or more. LTSA-MD has increased significantly in recent years, particularly among younger populations and women [8]. MDs also reduce workplace productivity through absenteeism and presenteeism [11].

The primary reason for LTSA is MD, which can have both positive and negative impacts on individuals and workplaces [9]. For instance, LTSA can facilitate recovery from illness and delay dismissal from work; however, it can negatively impact both quality of life and work life, leading to productivity loss and burdening of colleagues [9]. Therefore, sickness absences represent a significant public health and economic problem worldwide. Before the coronavirus disease 2019 (COVID-19) pandemic, the number of workers with LTSA due to MD (LTSA-MD) was increasing in Japan (among public workers) and in Western countries [8,10,12,13]. In Japan, LTSA-MD among public servants was reported at rates ranging from 495 to 780.6 per 100,000 employees [9].

The COVID-19 pandemic significantly impacted the physical and mental health of the general population [14]. Systematic reviews and meta-analyses conducted in the initial phase (up to 2020) revealed significant levels of psychological distress associated with the pandemic [15–17], including high rates of anxiety, depression, post-traumatic stress disorder, and stress in various countries [17]. In addition, healthcare workers and others reported high levels of fear [15]. Worsening depressive and anxiety symptoms were observed during the first 2 months of the pandemic, showing a linear relationship with the increasing number of reported COVID-19 cases and the rising stringency of governmental measures [16]. Meta-analyses during the early to middle (up to 2021) stage of the COVID-19 pandemic revealed a prevalence of mental health issues ranging from 20% to 36% and a higher prevalence of psychophysiological stress among healthcare workers than that among the general population [18]. LTSA due to mental health increased substantially among National Health Service staff in the United Kingdom (UK) during the first wave of the COVID-19 pandemic [19], and a similar trend was reported for overall mental illness-related absences among healthcare workers in the UK during the pandemic, with an increase observed compared to pre-pandemic levels [20]. Thus, the COVID-19 pandemic negatively impacted the mental health of workers across various occupational groups, including healthcare workers and public office employees. In particular, public office workers experienced a significant increase in LTSA-MD during the pandemic [12], likely due to unique stressors such as heightened job demands and increased public scrutiny. However, the effects of the pandemic on LTSA-MD among non-public office workers remain poorly understood.

The COVID-19 pandemic has also accelerated the adoption of remote work (RW) by employers and workers [21,22]. The adoption of RW surged during the pandemic, with organizations globally transitioning to teleworking to ensure business continuity. For instance, Park et al. reported that larger firms in South Korea adopted RW more effectively than smaller enterprises due to resource disparities [23], while Baudot and Kelly highlighted a significant shift in RW preferences in the United States, driven by perceived productivity gains during the pandemic [24]. However, RW has positively and negatively impacted workers’ health outcomes. Positive impacts of RW include improved family and work integration, increased productivity, greater job satisfaction, better self-rated health, enhanced quality of life and well-being, and reduced fatigue and role-related stress [25–30]. Conversely, some studies demonstrated the negative effects of RW on mental and physical health [31], such as decreased physical activity [32], reduced current and future health quality and well-being [33], increased emotional exhaustion, and increased loneliness [34]. Previous systematic reviews on the relationship between RW and worker health have shown conflicting results and weak evidence [35–38]. These studies are predominantly from Western contexts, and there is a notable lack of research investigating this relationship in Japan. Furthermore, there is a paucity of reports on the effects of RW on LTSA-MD trends during the COVID-19 pandemic.

We hypothesized that the number of non-public workers with LTSA-MD would increase during the COVID-19 pandemic compared with that before the pandemic, and that adopting RW would reduce the number of newly occurring LTSA-MD cases. Therefore, the aim of this study was to ascertain whether there was an increase in the number of workers with LTSA-MD and to assess the impact of RW on newly occurring LTSA-MD cases.

## Materials and methods

### Study design and participants

Based on the business companies’ information data obtained from the Japan Organization of Occupational Health and Safety (JOHAS) in September 2020, we mailed a request for cooperation in a web-based survey to 2,552 offices of companies with 150 or more workers in Osaka Prefecture, soliciting their responses. Osaka Prefecture was chosen as the study region due to its high population density, diverse industrial structure, and significant impact from the COVID-19 pandemic. The list of eligible companies was obtained from JOHAS and included all registered offices with 150 or more employees. No additional exclusions or stratifications were applied to the sampling process. The target population consisted of non-public sector workers from diverse industries, including manufacturing, healthcare, and services. These factors make Osaka a representative and relevant region for examining the interaction between RW adoption and LTSA-MD trends among non-public sector workers during the pandemic. The survey was conducted using a web-based platform with robust security measures to ensure the confidentiality and integrity of the data. Specifically, all data were encrypted during transmission using Hyper Text Transfer Protocol Secure (HTTPS) protocols and stored on a secure, password-protected server accessible only to authorized personnel. Additionally, a regular backup system was implemented to prevent data loss and ensure data availability. We conducted a cross-sectional survey and sent out reminders with a QR code and the URL of the questionnaire site to all 2,552 offices twice: on December 10, 2021, and January 13, 2022. Participants were informed that their participation was voluntary, and they provided written informed consent before completing the questionnaire. All data were stored only in our database, and the participants’ employers or the institutions did not have access to the data or know who had participated in the study. In this study, all data were accessed between January 15, 2022, and March 31, 2022. This study was approved by the Medical Research Ethics Review Committee of the Japan Labor Health and Safety Organization (registration number: 2021-04) and the Osaka City University Medical Research Ethics Review Committee (approval number: 2021-131). This study followed the Strengthening the Reporting of Observational Studies in Epidemiology (STROBE) reporting guideline to ensure comprehensive and transparent reporting [39]. Specifically, the study design, objectives, population, data collection methods, and analytical strategies were clearly defined and reported. Missing data handling and response rates were explicitly addressed, following the STROBE checklist. Additionally, robust data security measures were implemented to protect the confidentiality and integrity of the observational research data, including encryption during transmission, anonymization, secure storage, and regular backups.

### Data and statistical analysis

The questionnaire was conducted as a web-based survey with a focus on collecting aggregated office-level data. The questionnaire consisted of five main sections, each with predefined multiple-choice or short-answer formats. The content was as follows: 1) Whether the office had adopted RW practices during the COVID-19 pandemic (Yes/No); 2) the type of business activities, with predefined categories such as agriculture, manufacturing, healthcare, construction, wholesale, retail, and others; 3) the total number of full-time employees at the office; 4) the number of workers with LTSA-MD between April 1, 2019, and March 31, 2020 (pre-pandemic: fiscal year [FY] 2019), and between April 1, 2020, and March 31, 2021 (post-pandemic: FY 2020); and 5) the diagnosis of MDs among workers on LTSA, based on medical certificates. The diagnoses included adjustment disorder (AD), depressive disorder (DEP), bipolar disorder, schizophrenia, anxiety disorder, obsessive-compulsive disorder, sleep disorder, alcohol dependence, and other conditions. Employers are typically required to submit medical certificates issued by attending physicians when their employees take a leave of absence. These certificates provide the diagnosis determined by the physician, ensuring that the data reflect medical expertise rather than self-reported information. LTSA was defined as a period of sickness absence lasting 30 days or more. These predefined categories and structured questions were designed to ensure clarity and relevance for the survey participants. The difference in the number of new workers with LTSA-MD, LTSA-AD, and LTSA-DEP between FY 2020 and FY 2019 were calculated, divided by the number of employees in the office to evaluate the office size. Subsequently, the number of LTSA cases per 100 employees was calculated. An independent t-test was used to compare offices with and without an RW model, as it is appropriate for comparing means between two groups. To minimize the risk of Type I errors arising from multiple comparisons, the Bonferroni correction was applied, adjusting the significance level to 0.0167. The arithmetic expression was as follows: (the number of new workers with LTSA-MD, LTSA-AD, and LTSA-DEP in FY 2020 −  the number of new workers with LTSA-MD, LTSA-AD, and LTSA-DEP in FY 2019)/ (the number of employees in the office) ×  100. The effect size, Cohen’s *d*, was calculated using G-Power version 3.1 [40], and the value of power (1−β) was evaluated as 0.80, with α error =  0.05. The effect size was calculated to quantify the magnitude of group differences in LTSA-MD. It was computed as the difference between the means of the two groups divided by the pooled standard deviation. This standardized measure provides an effect size independent of sample size, allowing for meaningful comparison across studies. Additionally, the calculated effect size serves as a reference for future research, particularly for determining the necessary sample size to achieve adequate statistical power.

Statistical analyses were performed using IBM SPSS version 26.0 (IBM Corp., Armonk, NY, USA). Two-sided tests were conducted with statistical significance set at 0.05.

## Results

Of the 2,552 offices, 263 were included in the analysis (response rate: 10.3%). As shown in Table 1, job types included manufacturing (28.9%); healthcare and welfare (17.1%); wholesale and retail trade (16.7%); information, communication, and transportation (7.2%); construction (6.8%); and education and learning support (4.9%). Regarding the number of regular office workers, 16.3% had fewer than 50, 26.2% had 50–150, 37.3% had 151–300, 11% had 301–500, 6.5% had 501–1000, and 2.7% had more than 1001 workers. In total, 129 offices (49%) adopted an RW system, whereas 134 (51%) did not. The chi-squared test revealed no significant differences in the adoption rate of RW among offices of different sizes. Office sizes ranged from small to large, with no specific size group showing a higher propensity to adopt RW. In addition, Fisher’s exact test revealed no significant differences in the adoption rate of RW among offices in different business sectors, which included manufacturing, healthcare, and service industries, among others (Table 1). These findings indicate that the adoption of RW was not strongly associated with office size or sector during the pandemic.

**Table 1:** Office characteristics and RW adoption.

Business Sectors	No. (%)	RW(+)	RW(−)
Medical Welfare	45 (17.1)	8	37
Wholesale and Retail Trade	44 (16.7)	28	16
Education and Learning Support	13 (4.9)	5	8
Finance and Insurance	5 (1.9)	1	4
Construction	18 (6.8)	11	7
Accommodation and Drinking Services	8 (3)	6	2
“Information and Communications, Transportation and Postal Services "	19 (7.2)	6	13
Lifestyle-related Services and Amusements	6 (2.3)	5	1
Manufacturing	76 (28.9)	42	34
Professional and Technical Services	10 (3.8)	9	1
Real Estate	2 (0.8)	2	0
Others	17 (6.5)	6	11
Total	263 (100)	129	134
**Regular workers at the office**	**No. (%)**	**RW(+)**	**RW(−)**
50 >	43 (16.3)	23	20
50-150	69 (26.2)	35	34
151-300	98 (37.3)	45	53
301-500	29 (11)	12	17
501-1000	17 (6.5)	10	7
1001 ≦	7 (2.7)	4	3
Total	263 (100)	129	134

RW: remote work, No significant difference by Chi-square test and fisher's exact test

**Fig 1:**
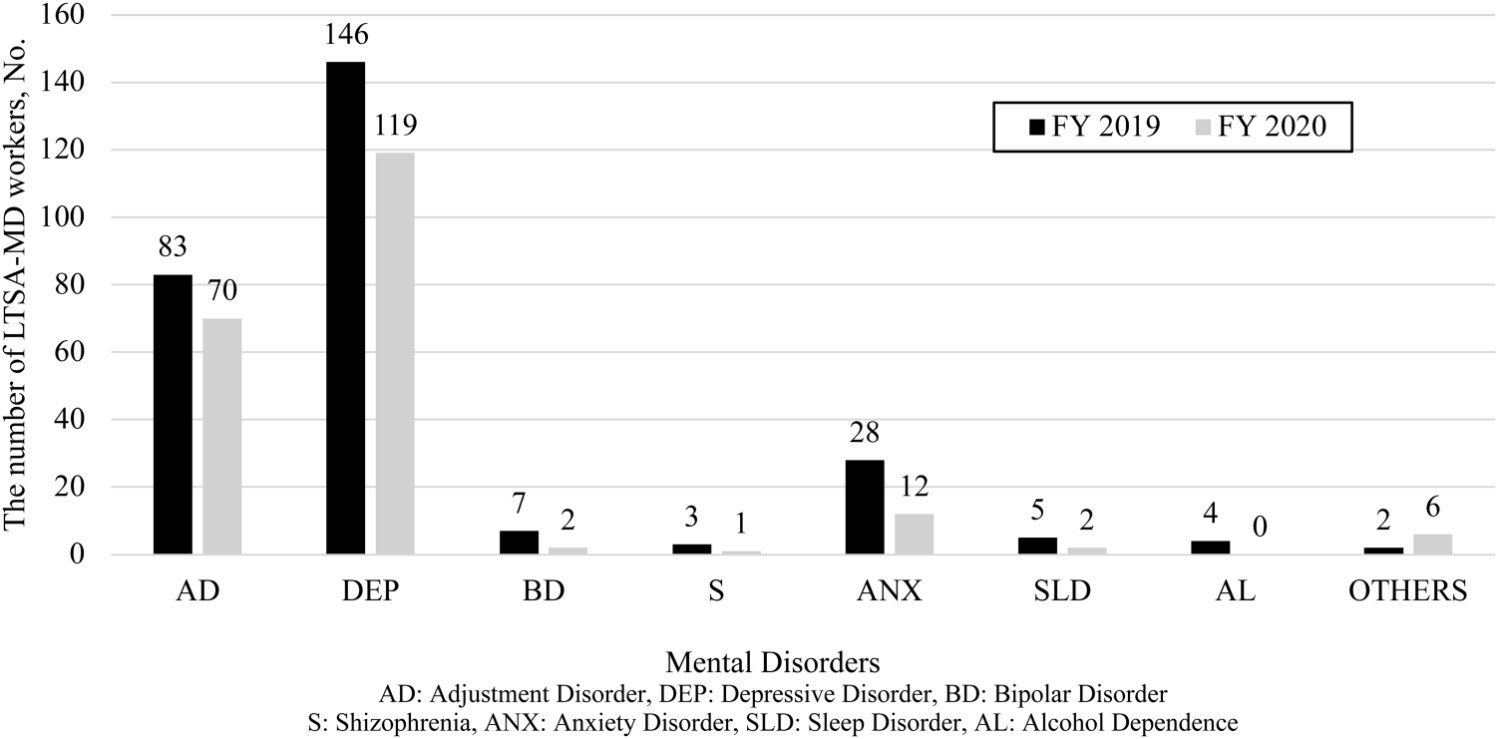
Newly occurring LTSA-MD cases in workers for over a month.

Fig 1 shows the number of workers newly experiencing LTSA-MD for 30 days or more, with 278 workers in FY 2019 and 212 workers in FY 2020. DEP was the most prevalent disorder, followed by AD and anxiety disorders. There were no cases of LTSA due to obsessive-compulsive disorders reported annually. The number of workers experiencing LTSA-MD nominally decreased from FY 2019 to FY 2020. However, no statistical analysis was conducted to determine the significance of this change, and thus no conclusions regarding statistical differences can be drawn (Fig 1).

Table 2 shows the differences in LTSA-MD, LTSA-AD, and LTSA-DEP between FY 2020 and FY 2019 for offices with and without an RW model, as calculated using an independent t-test. No significant differences were found between the following conditions: LTSA-MD (t (261) =  0.909, p =  0.364) with a Cohen’s d of 0.11, indicating a small effect size; LTSA-AD (t (261) =  0.84, p =  0.402) with a Cohen’s d of 0.1, indicating a small effect size; and LTSA-DEP (t (261) =  1.028, p =  0.305) with a Cohen’s d of 0.13 (Table 2). These small effect sizes suggest that the differences observed between offices with and without an RW model were minor, indicating that RW adoption may not have had a strong impact on LTSA-MD, LTSA-AD, or LTSA-DEP during the study period.

**Table 2:** Difference in the number of LTSA between FY 2020 and FY 2019.

	RW	NO	Mean (SD)	P
Difference between FY 2020 and FY 2019 on LTSA-MD	RW(−)	134	−0.0046 (1.7339)	.364
	RW(+)	129	−0.3332 (3.7931)	
Difference between FY 2020 and FY 2019 on LTSA-AD	RW(−)	134	−0.0583 (0.7076)	.402
	RW(+)	129	−0.201 (1.8286)	
Difference between FY 2020 and FY 2019 on LTSA-MDD	RW(−)	134	0.188 (2.0505)	.305
	RW(+)	129	−0.0462 (1.6069)	

SD: standard deviation, RW: remote work, LTSA: Long-Term Sickness Absence, MD: Mental Disorder, AD: Adjustment Disorder, DEP: Depressive Disorder, FY: Fiscal Year

## Discussion

The aim of this study was to explore trends in LTSA-MD and the impact of RW on new occurrences of LTSA-MD among Japanese non-public workers during the COVID-19 pandemic. Our findings revealed that the number of workers with LTSA-MD from FY 2019 to FY 2020 nominally decreased during the COVID-19 pandemic compared with that before the pandemic, although the difference was not significant. Additionally, no significant difference was found in the number of new LTSA-MD cases between offices with and without an RW model during the COVID-19 pandemic compared with that before the pandemic.

### Trends in the number of workers with LTSA-MD

Previous longitudinal studies have shown that high job demands [41], low job control, high job strain [41,42], and a combination of high strain and low support [43] are associated with an increased risk of LTSA-MD among workers. In our previous study conducted during the COVID-19 pandemic, we found no relationship between job demands, job control, anxiety, and depressive symptoms [44]. However, we found that variance in workload, job future ambiguity, and contact with COVID-19 patients were associated with anxiety or depressive symptoms and that social support from coworkers helped reduce anxiety and depressive symptoms among non-medical workers [44]. These findings suggest that self-perceived occupational stress during the pandemic was influenced by unique pandemic-specific factors, such as workload fluctuations and uncertainties about the future. While this study did not directly measure changes in occupational stress over time, these results provide indirect evidence that shifts in workplace stressors during the pandemic may have contributed to the observed nominal decrease in LTSA-MD cases. Future studies should directly examine the temporal dynamics of occupational stress and LTSA-MD to better understand this relationship.

Previous meta-analyses and systematic reviews have indicated that the COVID-19 pandemic affected workers’ mental health in its early stages (up to 2020) [15–18]. However, in the present study, the number of workers with LTSA-MD nominally decreased during the COVID-19 pandemic compared with that before the pandemic. A previous study in Norway evaluated the prevalence of current MD using validated diagnostic methods in cross-sectional random sample data collected from January 28 to March 11, 2020 (n =  563, 15.4%), March 12 to May 31, 2020 (n =  691, 9.0%), June 1 to July 31, 2020 (n =  530, 14.3%), and August 1 to September 18, 2020 (n =  370, 11.9%). Results showed that the prevalence of MD remained constant or decreased slightly throughout the pandemic period [45]. Before the pandemic, we reported a 10-year decreasing trend in LTSA-MD among Japanese public servants, observed over a 3-month period, from 2009 to 2018 [9]. A cross-sectional study among Japanese workers demonstrated that approximately 19% of workers experienced sickness presenteeism during the COVID-19 pandemic [46]. Up to 2020, the pandemic may not have increased the number of workers with MD, as observed in a Norwegian study [40] that reported a stable or slightly decreasing prevalence of MD throughout the pandemic. This trend aligns with our finding of a nominal decrease in LTSA-MD cases during the COVID-19 pandemic. One potential explanation for this trend is that workers may have avoided absenteeism due to increased job insecurity or workplace expectations, and continued to work despite mental health challenges, a behavior commonly referred to as presenteeism. A Japanese cross-sectional study showed that approximately 19% of workers experienced sickness presenteeism during the pandemic [46]. This behavior could have masked the true burden of MD, as workers chose to remain in their roles rather than taking LTSA. Our findings suggest that working with presenteeism may have contributed to the nominal reduction in LTSA-MD observed in this study. Future research should investigate the long-term impacts of presenteeism on workers’ mental health and LTSA-MD trends. The absence of cases for certain disorders, such as obsessive-compulsive disorders, may reflect potential underreporting due to stigma associated with these conditions or diagnostic challenges in workplace settings. This limitation highlights the need for more comprehensive data collection in future studies to better understand the full scope of LTSA-MD.

### Newly occurring LTSA-MD cases in workers by RW status

The previous four systematic reviews on the relationship between RW and workers’ health yielded conflicting results based on weak evidence [35–38]. RW positively and negatively impacts workers’ health outcomes. In this study, while RW provided flexibility and protection against infection, its benefits were likely offset by increased work-home interference and emotional exhaustion during the pandemic. This balance may explain the nominal reductions in LTSA-MD observed in RW offices, which were not statistically significant. However, some studies have reported adverse effects. Allen et al. indicated that the blurring of physical and organizational boundaries between work and home could adversely affect an individual’s mental and physical health owing to extended hours, lack of clear delineation between work and home, and inadequate support from organization [31]. A cross-sectional survey in Japan showed that remote employees had lower levels of physical activity and extended periods of inactivity during work hours compared to on-site employees [32]. A cross-sectional study in the United States reported that COVID-19-related employment changes were associated with increased sitting and screen time, and sedentary time is consistently adversely associated with current and future health and well-being [33]. Wang et al. showed a correlation between high workload, increased work–home interference, increased emotional exhaustion, and loneliness [34].

Conversely, employees working from home reported an overall lower risk of developing poor health, compared with those working from the office [26], and RW has been reported to have a positive impact on improved family and work integration, productivity, job satisfaction, quality of life, and reductions in fatigue and role-related stress [25,27,28]. During the pandemic, the number of employees working from home increased drastically, potentially encompassing millions of employees worldwide who were temporarily engaged in RW [21]. Ervasti et al., in a cohort study among 24,299 Finnish public sector employees, found that workers engaged in RW during the pandemic perceived their self-rated health as better than that of those with on-site jobs in 2020 [30]. Kaltiainen et al. conducted a longitudinal study in Finland, demonstrating that non-teleworkers experienced less favorable changes in occupational well-being and more instances of burnout, compared with teleworkers [29]. The authors concluded that teleworking might have supported certain working conditions, such as introducing new work practices, increased autonomy in the workplace, and opportunities for learning and skills acquisition, which foster occupational well-being, such as work engagement [29].

In the present study, offices that adopted RW had a nominal reduction in the proportion of newly occurring LTSA-MD, LTSA-AD, and LTSA-DEP cases, compared to those that did not adopt RW during the COVID-19 pandemic, with no significant differences. Cohen’s *d* was calculated, indicating a small effect size. RW enables employees with MD to work from home, potentially reducing LTSA by allowing workers to maintain productivity and avoid absenteeism. However, this flexibility may also promote presenteeism, delaying recovery and increasing the risk of long-term mental health complications. In contrast, in settings where physically attending the workplace is the only option, employees with MD may be more likely to require LTSA. A report indicated that regularly commuting to the workplace was associated with a higher risk of infection [47]. We previously reported that reducing time spent outside could prevent anxiety [48]. A cross-sectional study among Japanese workers demonstrated that the issue of sickness presenteeism became more prominent during the COVID-19 epidemic [46]. We hypothesize that the effects of RW, such as protection against COVID-19 infection and anxiety [47,48] and ability to continue working with presenteeism [46], are specific to the pandemic. During COVID-19, the pandemic-specific positive and negative effects of RW likely counteracted each other. While RW reduced anxiety and provided a means for work continuity, increased emotional exhaustion and the blurring of work-home boundaries may have diminished these benefits. This dynamic likely explains the lack of significant differences observed in LTSA-MD between RW and non-RW offices. This lack of significant differences suggests that RW alone may not be sufficient to mitigate LTSA-MD trends. Instead, implementing comprehensive workplace health policies, including targeted mental health interventions and fostering a supportive organizational culture, may be more effective in improving worker well-being. In the present study, although no significant differences were found, offices that adopted RW had a nominal reduction in the number of LTSA-DEP cases among the workers, while offices that did not adopt RW showed a nominal increase in this number during the COVID-19 pandemic compared with that before the pandemic. The number of patients with DEP reportedly increased during the pandemic [49], and RW might have a protective effect on workers newly found to have LTSA-DEP. However, the effects of adopting RW for this population remain unclear. Future research should focus on longitudinal analyses to understand the long-term impact of RW on LTSA-MD, examine the role of presenteeism in RW settings, and explore how organizational policies and support systems can enhance the positive effects of RW while mitigating its negative impacts.

### Strengths and limitations

This study has both strengths and limitations. Collecting accurate information from a wide range of non-public workers experiencing LTSA-MD was challenging, as non-public companies and offices fear a downturn in their public image. However, to our knowledge, this is the first study to clarify the trends of LTSA-MD among non-public sector workers before and during the COVID-19 pandemic in all Osaka areas, which is a significant strength. The limitations of this study were as follows. First, the response rate was low. Because of the pandemic, a web-based survey was used to avoid contact with the respondents as much as possible. Requests for cooperation were sent to 2,552 offices belonging to companies with 150 or more workers in Osaka Prefecture, and valid responses were received from 263 offices (response rate: 10.3%) for analysis. This low response rate may have introduced response bias, potentially limiting the generalizability of the findings. The use of a web-based questionnaire, rather than a paper-based survey, during a time of difficult coordination in offices due to the pandemic, might have affected participation. Only offices that were interested participated in the survey, and the survey was not completed by the offices with an increased number of workers with MD. As this study focused on the Osaka Prefecture, findings may not be directly generalizable to regions with different demographic or industrial characteristics. Second, a selection bias exists. Although we requested cooperation from offices of companies with 150 or more workers, approximately 6% of the responses came from companies with fewer than 150 workers. In fact, approximately half of the offices had fewer than 150 employees. This discrepancy could be attributed to organizational changes during the COVID-19 pandemic, such as workforce reductions or restructuring, which might have caused some companies to fall below the 150-employee threshold. This selection bias may have influenced the findings, particularly if smaller offices exhibited different LTSA-MD trends or RW adoption rates compared to larger offices. Future studies should consider methods to account for such changes, such as cross-validating workforce data with updated records or implementing stratified sampling approaches to ensure representativeness. Third, the relationship with the COVID-19 pandemic was unclear; however, the number of employees may have fluctuated significantly due to workforce reductions or restructuring during the pandemic. These fluctuations may have masked the true prevalence of LTSA-MD or influenced the adoption of RW, potentially confounding the results. This could affect the interpretation of the results, and the representativeness of the results should be considered when interpreting them. Fourth, the data were self-reported, potentially leading to response bias and misclassifications. To mitigate this, the survey was designed with clear and concise questions, and responses were collected anonymously to encourage honesty. However, the inherent limitations of self-reported data remain, and future studies should consider validating findings through objective measures, such as medical records or third-party verification. Fifth, while we inquired whether RW was adopted during the survey, information on whether it was adopted pre-pandemic was lacking and this study does not capture whether individual workers of the companies adopted RW. This represents a significant gap in understanding the full impact of RW. Addressing this gap requires detailed data on the number of workers engaging in RW, its frequency, the transition of its adoption in each office, and pre-pandemic data to enable a more comprehensive evaluation of RW’s long-term impact. Additionally, this study was not designed to track individual workers’ employment statuses over time. As such, we cannot determine whether the observed reduction in LTSA cases reflects true decreases in prevalence or changes in workforce composition. Sixth, a request was mailed to the human resources and labor management department of each company; however, we did not collect specific information on whether a single individual or multiple employees were involved in completing the questionnaire. Seventh, our study is limited by its reliance on aggregated data collected over pre-defined fiscal years, which may not fully capture the temporal dynamics of mental health outcomes during the COVID-19 pandemic. Additionally, the use of basic statistical analyses, such as mean difference tests, limits the ability to explore complex relationships and long-term trends. However, our findings provide valuable insights into immediate workplace-level trends in mental health challenges during the pandemic and contribute to the field of disaster psychiatry. Eighth, this study did not collect monthly data on LTSA occurrences and instead focused on annual trends before and during the COVID-19 pandemic. Monthly data would provide valuable insights into the dynamics of LTSA and RW adoption, particularly during critical phases of a crisis. Future research should prioritize incorporating monthly data collection to better understand these dynamics.

## Conclusion

This study demonstrated that the number of non-public workers with LTSA-MD did not increase during the COVID-19 pandemic. This trend may reflect the impact of workplace policies and mental health interventions implemented during this pandemic period. Measures such as flexible work arrangements, stress management programs, and RW adoption, may have helped mitigate the impact of the pandemic on workers’ mental health. There was no significant difference in the number of new workers with LTSA-MD between offices with and without an RW model during the COVID-19 pandemic compared with that before the pandemic. Our study provides valuable insights into the relationship between RW and LTSA-MD, offering preliminary evidence of a potential protective effect of RW against LTSA-DEP. However, the mechanisms by which RW influences mental health outcomes, including its potential to reduce LTSA-DEP, remain unclear. Future investigations should focus on longitudinal analyses to explore the long-term impact of RW on LTSA-MD and examine the interplay between RW, workplace policies, and individual mental health outcomes. Such research should also consider specific organizational interventions and their effectiveness in supporting workers with MDs in both RW and on-site environments.

## Supporting information

S1 File Questionnaire. (PDF)
