# Supplementary material for: Remote work and long-term sickness absence due to mental disorder trends among Japanese workers pre/post COVID-19
Source: PLoS One. 2025 Mar 12;20(3):e0319825. doi: 10.1371/journal.pone.0319825 (PMC11902284; doi:10.1371/journal.pone.0319825)
Supplement: S1 File — (PDF) [file pone.0319825.s001.pdf]

本調査に協力していただくことに同意いただけますか？ はい ・ いいえ

はいに〇を付けていただいた方は、以下の質問すべてにご記入下さい。

御社（貴事業所）の概要についてお伺いします

1) 事業内容　　農業・林業　漁業　工業　建設業　製造業　電気・ガス・水道業
情報通信業運輸業・郵便業　卸売業・小売業　金融業・保険業　不動産業
専門・技術サービス業　宿泊業・飲酒サービス業　生活関連サービス業・娯楽業
教育・学習支援業　医療福祉　その他（　　　　）

2) 本社支社の区分　　本社　支社・支店　その他（　　　　）

3) 従業員数　事業所：フルタイム（　　　）人、非正規（　　　）人、企業全体（　　　）人

現在のメンタルヘルス不調者に対する支援体制、労務規程についてお伺いします

リモートワークを導入していますか？　　あり・なし

メンタルヘルス不調者の実態に関してお伺いします

1) 2019年4月1日～2020年3月31日（beforeコロナ）までの約1年間で新たに発生した、メンタルヘルス不調により30日以上休業した職員の人数　　（　　　）人

2) 2019年4月1日～2020年3月31日までの約1年間（beforeコロナ）で新たに発生した、メンタルヘルス不調により30日以上休業した職員の診断書傷病名、年齢、休職時期と採用時の属性

	合計
適応障害	人
うつ病・(抑)うつ状態	人
双極性障害・躁うつ病	人
統合失調症・幻覚妄想状態	人
神経症・自律神経失調症	人
不安障害・パニック障害	人
強迫性障害	人
不眠症・睡眠障害	人
アルコール依存症	人
広汎性発達障害・自閉スペクトラム症	人
その他（　　　　）	人

3) 2020年4月1日～2021年3月31日までの約1年間（withコロナ）で新たに発生した、メンタルヘルス不調により30日以上休業した職員の人数　　（　　　）人

4) 2020年4月1日～2021年3月31日までの約1年間（withコロナ）で新たに発生した、メンタルヘルス不調により30日以上休業した職員の診断書傷病名、年齢、休職時期と採用時の属性

	合計
適応障害	人
うつ病・(抑)うつ状態	人
双極性障害・躁うつ病	人
統合失調症・幻覚妄想状態	人
神経症・自律神経失調症	人
不安障害・パニック障害	人
強迫性障害	人
不眠症・睡眠障害	人
アルコール依存症	人
広汎性発達障害・自閉スペクトラム症	人
その他（　　　　　　　）	人

最後に記載漏れがないかご確認ください。御協力いただきありがとうございました。
